# A case report of pregnancy in a patient with common variable immunodeficiency emphasizing the need for personalized immunoglobulin replacement

**DOI:** 10.1097/MD.0000000000012804

**Published:** 2018-11-02

**Authors:** Ewa Więsik-Szewczyk, Karina Jahnz-Różyk

**Affiliations:** Department of Internal Medicine, Pneumonology, Allergology, and Clinical Immunology, Central Clinical Hospital of the Ministry of National Defense, Military Institute of Medicine, Warsaw, Poland.

**Keywords:** hyaluronidase, pregnancy, primary antibody deficiency, subcutaneous administration

## Abstract

**Rationale:**

Subcutaneous immunoglobulin administration facilitated by recombinant human hyaluronidase is a new mode of immunoglobulin replacement. It has been approved for treatment in primary and secondary antibody immunodeficiency. To date, it has not been reported in the literature as therapy of choice during pregnancy.

**Patient concerns:**

We report a 31-year-old woman with common variable immunodeficiency (CVID) followed during her first pregnancy.

**Diagnoses:**

The patient had a history of increased susceptibility to infections and autoimmune phenomena. From diagnosis at the age 29, she received IVIg replacement with partial response to treatment. Due to medical indications and lack of venous access, we had to search for another mode of application. The patient refused traditional, weekly conventional subcutaneous immunoglobulin (SCIg) administration.

**Interventions:**

Immunoglobulin replacement therapy was successfully continued during pregnancy after the IV route was replaced with subcutaneous administration facilitated by recombinant human hyaluronidase. The frequency of infusions was every 3–4 weeks.

**Outcomes:**

The treatment was effective and well tolerated by the patient who continued it after delivery. Dosage and the schedule of infusions provided sufficient immunoglobulin G (IgG) levels for the newborn baby.

**Lessons:**

The presented CVID case illustrates that the selection of the mode of immunoglobulin administration has to be a shared decision, which considers both patient preferences and medical needs. This approach is especially important for the pregnancy period. The case shows that the switch from IVIg to fSCIg can be a management option during pregnancy.

## 1 Introduction

Common variable immunodeficiency (CVID) is the most prevalent and clinically significant primary antibody deficiency. Despite being a hereditary condition, CVID often manifests its first signs in the third decade of life, the time when women decide on motherhood.^[1]^ Lifelong immunoglobulin replacement therapy in primary antibody deficiencies offers effective protection against recurrent infections. Thus, more patients can lead a social life, accomplish education, marry, and experience parenthood. Women with CVID consider pregnancy to be a normal part of their life. An Internet-based survey performed in the United States (US) revealed that in women with primary immune deficiencies, spontaneous pregnancy loss for first and second pregnancies did not differ from the US general population.^[1]^ Therapeutic, polyclonal immunoglobulin G (IgG) can be administered intravenously (intravenous immunoglobulin [IVIg]), subcutaneously (subcutaneous immunoglobulin [SCIg]), and subcutaneously facilitated by recombinant human hyaluronidase (hyaluronidase facilitated subcutaneous immunoglobulin [fSCIg]).^[2–6]^ In some countries similarly to Poland, IVIg administration is available only in hospitals, conventional SCIg and fSCIg can be self-administered by patients at home. Each mode has its own advantages and disadvantages.^[2]^ Conventional SCIg seems to be preferred by many patients and doctors. Venous access is not required, the need for premedication reduced, mean serum IgG levels are stable and systemic adverse side effects extremely rare, with hospital or office visits reduced.^[3]^ On the other hand, conventional SCIg requires frequent administration, typically every week, with small volumes injected in multiple sites and is burdensome for some patients. fSCIg is a compromise between the SCIg and IVIg modality. It can be given subcutaneously but less frequently than conventional SCIg. The administration of recombinant human hyaluronidase specifically and temporarily cleaves hyaluronate in extracellular matrix and locally increases the movement of fluid and its contact with lymphatic vascular space, facilitating the absorption of molecules as large as immunoglobulins.^[4]^ This allows to reduce the frequency of immunoglobulin administration to 3- or 4-weekly and increases volumes administered in 1 injection site. Data from the phase III clinical trial and real life data proved the modality's efficacy and safety.^[5,6]^

During pregnancy in CVID, IgG supplementation has to be continued, to protect the mother and to be a source of IgG for the newborn, as IgG are actively transported across the placenta. Available data regarding optimal modes of administration and doses during pregnancy are limited,^[1,7–10]^ but there are suggestions that the demand for IgG increases with gestational age.^[11]^ In this report we discuss a woman with CVID switched from IVIg to fSCIg replacement during pregnancy. Written informed consent was obtained from the patient for publication of this report.

## 2 Case presentation

The patient was a 31-year-old woman diagnosed with CVID. She had chronic sinusitis at 20, and beginning at age 25 was repeatedly treated with antibiotics because of recurrent bronchitis. She smoked 20 cigarettes per day since she was 18. The patient did not receive prophylactic vaccination against influenza, pneumococci, or *Haemophilus influenzae*. She was referred to a clinical immunologist at 29 because of 2 episodes of severe pneumonia in the course of 1 year. She has significant vitiligo and a congenital hypoplastic left kidney. Her family history of chronic diseases was unremarkable. Testing at the Department of Immunology confirmed: a persistent deficiency of 3 main classes of antibodies: IgG 10 mg/dL (n = 600–1600), immunoglobulin M (IgM) 4 mg/dL (n = 40–230), absent immunoglobulin A (IgA), and absent isohaemagglutinins. Flow cytometry found an increased percentage of non-switched memory B cells (IgM++ IgD+ CD38+ CD27+ CD21+) 22% (3.3–12.8%), but lowered class-switched memory B cells (IgM– IgD– CD38+ CD27+ CD21+) –3.2% (4.0–22.1%). We excluded T-cell deficiency and human immunodeficiency virus (HIV) infection, by polymerase chain reaction analysis. Based on the long-term history of increased susceptibility to infection and results of laboratory tests we diagnosed the patient with CVID and qualified her for immunoglobulin replacement. In September 2015, she received the first IVIg which was continued regularly at doses of 0.5 to 0.6 g/kg/mo. In the course of treatment we achieved partial clinical response: there were no severe bacterial infections but recurrent bronchitis persisted. She needed repeated oral antibiotic cycles. A chest computerized tomography (CT) scan after 1 year of treatment revealed mild bronchiectases and interstitial lung inflammation. The IgG trough level was 710 mg/dL. Her body weight increased from 67 to 82 kg, body mass index increased from 25.22 to 30.86 before pregnancy. Moreover she had very difficult venous access. Despite education she did not stop smoking, did not go on a diet, or did not change her lifestyle. Due to difficult venous access, high demand for immunoglobulin (50 g every 3–4 weeks) and wear off effect on IVIg, we proposed changing to conventional SCIg treatment. In November 2016 she found she was pregnant and refused to change replacement modality for weekly home SCIg therapy. During pregnancy peripheral swelling made peripheral venous access impossible. In week 27 of pregnancy, after obtaining the patient's consent, we introduced fSCIg every 3 or 4 weeks. The treatment was well tolerated. Infusions were given into both thighs, simultaneously 25 g 10% IgG (250 mL) facilitated by 12.5 mL recombinant human hyaluronidase per site, at 300 mL/h infusion rate. The treatment schedule is presented in Table 1. At week 41, she was delivered of a healthy boy (2780 g/52 cm) by cesarean section indicated due to lack of progress in the first phase of delivery. Six days after delivery the baby's IgG level was 889 mg/dL. Postpartum, the mother continued fSCIg at the same dose. Now she is trained for home fSCIg administration. Her last IgG trough level was 729 mg/dL.

**Table 1:**
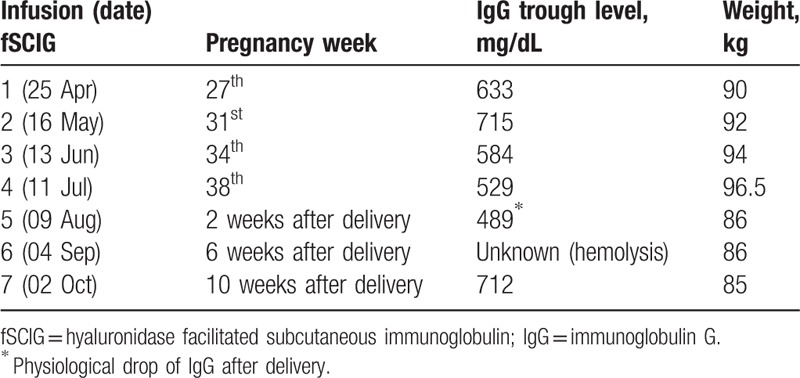
Schedule of subcutaneous immunoglobulin administration facilitated by recombinant human hyaluronidase during pregnancy and postpartum.

## 3 Discussion

We are not aware of any other reports of a switch from IVIG to fSCIG during pregnancy in CVID. We showed that fSCIg can be administered at the same dose and schedule as IVIG even in the third trimester of pregnancy (Table 1).

The presented CVID subject had been under IVIg treatment for 1 year. During her management and follow-up we had difficulties with venous access, we observed partial clinical efficacy and IVIg wear off effect. The patient required high IVIg volume, was obese and had an increased risk of thromboembolic events. The risk–benefit ratio for inserting a permanent vascular catheter versus introducing a new immunoglobulin administration modality in our opinion favored switching to fSCIg. The patient approved of the new technique. The treatment was biologically effective and well tolerated by the patient who continued the treatment after delivery. The applied Ig administration schedule provided a sufficient IgG level for the newborn baby.

Immunoglobulin replacement is essential for a pregnant woman with CVID. It protects the mother from infections and is a source of IgG for the infant, who receives immunoglobulin by placental transfer.^[7,8]^ There are data suggesting that IVIg efficiency decreases in the second and third trimester of pregnancy.^[11]^ SCIg can be a convenient mode of administration. In 2001, Gardulf et al^[9]^ evaluated the effect of rapid, SCIg infusions during 11 pregnancies in 9 women with primary antibody deficiencies. Weekly infusions were self-administered by the subjects at a dose of 0.1 g/kg/wk throughout the pregnancy. No adverse systemic reactions or pronounced, local tissue reactions were recorded during or after the >400 infusions. The 11 babies were healthy and were born after 38 to 42 weeks of uneventful gestation. The concentrations of maternal IgG at the time of delivery in the 4 women with CVID ranged from 600 to 830 mg/dL. The SCIg home-therapy regime was appreciated by the patients. If high immunoglobulin doses are required, even daily subcutaneous infusions are feasible.^[10]^

However, despite medical advantages SCIg might not be accepted by some patients. Danieli et al^[12,13]^ described a 36-year-old woman with CVID, a high risk pregnancy and severe adverse reactions to IVIg who refused SCIg and preferred to be treated intravenously. Our patient had been offered conventional SCIg treatment but found the weekly home self-infusions too burdensome. She presented a passive attitude and was averse to any change of therapy and the unplanned pregnancy compounded the therapeutic decisions even further. We could overcome this by introducing fSCIG at the day-case clinic.

Pregnancy in chronic disease is always a challenge for the patient, her family, and medical staff. We always recommend that patients plan pregnancies when optimal disease control and mode of immunoglobulin administration are established. Our patient's pregnancy was unplanned, occurring before her optimal disease control and mode of immunoglobulin supplementation was established. Initially we were not able to educate her in home self-therapy during pregnancy. This limited economic advantages of subcutaneous treatment. She changed her attitude after delivery and started self-infusions at home.

Based on our case report we would like to emphasize the importance of shared decision-making by medical staff and patients regarding selection of Ig replacement mode that takes into account medical needs and patient preferences. We would like to add fSCIG to the list of management options during pregnancy. We confirm that the shift from IVIg to fSCIG is safe and efficient during pregnancy, especially when conventional SCIg is not accepted by the patient and IVIg is contraindicated.

## Author contributions

**Conceptualization:** Ewa Więsik-Szewczyk, Karina Jahnz-Różyk.

**Data curation:** Ewa Więsik-Szewczyk.

**Formal analysis:** Ewa Więsik-Szewczyk.

**Supervision:** Karina Jahnz-Różyk.

**Writing – original draft:** Ewa Więsik-Szewczyk.

**Writing – review & editing:** Ewa Więsik-Szewczyk.

Ewa Więsik-Szewczyk orcid: 0000-0001-8509-4453.
